# Effects of Aerobic Exercise on Brain Age and Health in Middle-Aged and Older Adults: A Single-Arm Pilot Clinical Trial

**DOI:** 10.3390/life14070855

**Published:** 2024-07-08

**Authors:** An Ouyang, Can Zhang, Noor Adra, Ryan A. Tesh, Haoqi Sun, Dan Lei, Jin Jing, Peng Fan, Luis Paixao, Wolfgang Ganglberger, Logan Briggs, Joel Salinas, Matthew B. Bevers, Christiane Dorothea Wrann, Zeina Chemali, Gregory Fricchione, Robert J. Thomas, Jonathan Rosand, Rudolph E. Tanzi, Michael Brandon Westover

**Affiliations:** 1 Henry and Allison McCance Center for Brain Health, Massachusetts General Hospital, Boston, MA 02114, USAzhang.can@mgh.harvard.edu (C.Z.); ryan.tesh@mgh.harvard.edu (R.A.T.); hsun8@mgh.harvard.edu (H.S.); cwrann@mgh.harvard.edu (C.D.W.);; 2 Department of Neurology, Massachusetts General Hospital, Boston, MA 02114, USA; jjing@partners.org (J.J.);; 3 Harvard Medical School, Boston, MA 02115, USAmbevers@bwh.harvard.edu (M.B.B.); rthomas1@bidmc.harvard.edu (R.J.T.); 4 Marcus Institute for Aging Research, Hebrew SeniorLife, Boston, MA 02131, USA; 5 Department of Physical Therapy & Human Movement Science, Feinberg School of Medicine, Northwestern University, Chicago, IL 60611, USA; 6 Department of Neurology, Washington University School of Medicine in St. Louis, St. Louis, MO 63110, USA; 7 Department of Neurology, New York University Grossman School of Medicine, New York, NY 10016, USA; 8 Department of Neurology, Brigham and Women’s Hospital, Boston, MA 02115, USA; 9 Cardiovascular Research Center, Massachusetts General Hospital, Boston, MA 02114, USA; 10 Department of Psychiatry, Massachusetts General Hospital, Boston, MA 02114, USA; 11 Department of Medicine, Division of Pulmonary, Critical Care & Sleep, Beth Israel Deaconess Medical Center, Boston, MA 02215, USA

**Keywords:** sleep, exercise, intervention trial, brain health, EEG

## Abstract

Backgrounds: Sleep disturbances are prevalent among elderly individuals. While polysomnography (PSG) serves as the gold standard for sleep monitoring, its extensive setup and data analysis procedures impose significant costs and time constraints, thereby restricting the long-term application within the general public. Our laboratory introduced an innovative biomarker, utilizing artificial intelligence algorithms applied to PSG data to estimate brain age (BA), a metric validated in cohorts with cognitive impairments. Nevertheless, the potential of exercise, which has been a recognized means of enhancing sleep quality in middle-aged and older adults to reduce BA, remains undetermined. Methods: We conducted an exploratory study to evaluate whether 12 weeks of moderate-intensity exercise can improve cognitive function, sleep quality, and the brain age index (BAI), a biomarker computed from overnight sleep electroencephalogram (EEG), in physically inactive middle-aged and older adults. Home wearable devices were used to monitor heart rate and overnight sleep EEG over this period. The NIH Toolbox Cognition Battery, in-lab overnight polysomnography, cardiopulmonary exercise testing, and a multiplex cytokines assay were employed to compare pre- and post-exercise brain health, exercise capacity, and plasma proteins. Results: In total, 26 participants completed the initial assessment and exercise program, and 24 completed all procedures. Data are presented as mean [lower 95% CI of mean, upper 95% CI of mean]. Participants significantly increased maximal oxygen consumption (Pre: 21.11 [18.98, 23.23], Post 22.39 [20.09, 24.68], mL/kg/min; effect size: −0.33) and decreased resting heart rate (Pre: 66.66 [63.62, 67.38], Post: 65.13 [64.25, 66.93], bpm; effect size: −0.02) and sleeping heart rate (Pre: 64.55 [61.87, 667.23], Post: 62.93 [60.78, 65.09], bpm; effect size: −0.15). Total cognitive performance (Pre: 111.1 [107.6, 114.6], Post: 115.2 [111.9, 118.5]; effect size: 0.49) was significantly improved. No significant differences were seen in BAI or measures of sleep macro- and micro-architecture. Plasma IL-4 (Pre: 0.24 [0.18, 0.3], Post: 0.33 [0.24, 0.42], pg/mL; effect size: 0.49) was elevated, while IL-8 (Pre: 5.5 [4.45, 6.55], Post: 4.3 [3.66, 5], pg/mL; effect size: −0.57) was reduced. Conclusions: Cognitive function was improved by a 12-week moderate-intensity exercise program in physically inactive middle-aged and older adults, as were aerobic fitness (VO_2_max) and plasma cytokine profiles. However, we found no measurable effects on sleep architecture or BAI. It remains to be seen whether a study with a larger sample size and more intensive or more prolonged exercise exposure can demonstrate a beneficial effect on sleep quality and brain age.

## 1. Introduction

Sleep and brain health are tightly linked, with better sleep correlating with favorable objective and subjective measures of brain structure [1,2] and function [3,4,5]. Throughout the aging process, there is a progressive loss of “healthy” features of sleep macro- and micro-architecture, characterized by reductions in N3 sleep, sleep efficiency, and delta power. These alterations are more pronounced in certain neurological disorders, such as Alzheimer’s disease (AD) and Parkinson’s disease (PD). It is noteworthy that a substantial proportion, ranging from 40% to 60%, of individuals with PD report experiencing sleep disturbances [6]. These aging-associated sleep disturbances are independent risk factors that appear to contribute to and reflect both impaired cognitive function [7,8,9,10] and mortality in older adults [11,12]. There is a critical need for (1) practical and precise ways to track the effect of interventions that aim to improve brain health, and (2) non-pharmacological approaches to improve sleep and thus positively impact brain health.

Exercise is an attractive intervention of key interest, as accumulating evidence shows beneficial effects on both sleep quality and measures of cognitive health in older adults [13,14,15,16]. Previous work has suggested that exercise increases N3 sleep, which has been linked to brain aging [17] and fast-sigma power and reduces sleep fragmentation [18,19,20,21]. However, other studies have found minimal effects of exercise on N3 sleep [22,23]. In addition, the extent to which exercise improves cognitive function in older adults with dementia remains a topic of investigation [24,25]. Recent AAN (American Academy of Neurology), ACSM (American College of Sports Medicine), and WHO guidelines recommend that older individuals exercise 150–300 min weekly to maintain brain health [26,27]. However, the optimal frequency, intensity, time, and type (FITT) of aerobic exercise are debatable. Overall, the differences in exercise regimen design, target population, and sleep and cognitive assessments in previous exercise intervention trials leave open the question of whether and to what extent exercise is able to improve brain health.

Our team recently developed a brain health biomarker called “Brain Age” (BA) derived from sleep EEG and recordings [28]. BAI is the estimated BA minus chronological age (BAI = BA − CA), i.e., the difference between apparent (predicted) age and biological age. BAI is increased in individuals with neuropsychiatric (mild cognitive impairment or dementia) [29] and cardiometabolic diseases [28]. However, no study has evaluated whether BAI can be reduced through interventions that improve brain health. In addition, the practical challenges associated with the large-scale and longitudinal application of BAI within lab settings, including high costs, night-to-night variability of sleep, and the first-night effect in sleep laboratory testing, pose limitations. Despite recent advancements, such as the introduction of the sleep headband Dreem 2 aimed at improving sleep monitoring with fewer interferences mentioned above in home settings, the comparability of BAI computed from home sleep EEG recordings (e.g., captured by Dreem2) with BAI determined from the gold standard polysomnography (PSG) method for sleep monitoring remains uncertain.

Here, we conducted an exploratory study (1) to evaluate whether the combination of a home sleep headband and BAI was able to track brain health in healthy middle-aged and older adults long term; and (2) to investigate whether a 12-week, 150 min weekly moderate-intensity aerobic exercise regimen designed to improve aerobic fitness could improve brain health, as reflected by an improvement in cognition, a reduction in BAI, and the enhancement of key features of sleep macro- and micro-architecture.

## 2. Methods

### 2.1. Study Design and Participants

**Figure 1:**
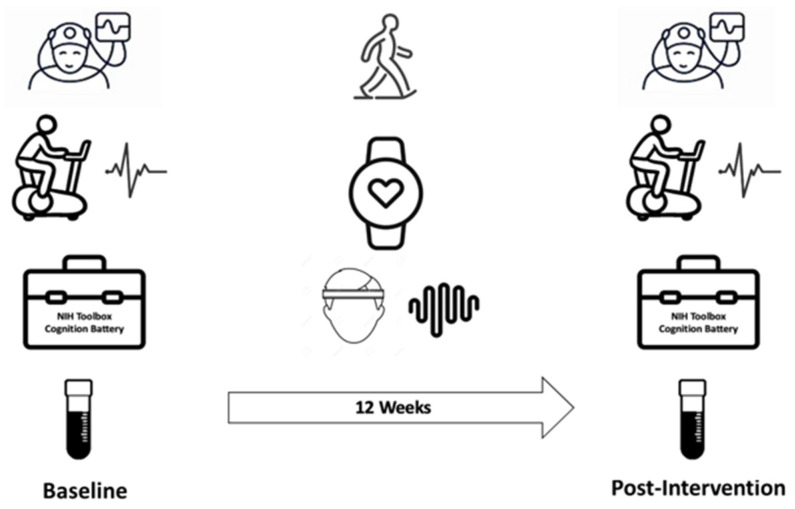
Study design. In the figure, left/right column pictures from top to bottom are polysomnography (PSG), cardiopulmonary exercise testing (CPET), NIH toolbox cognition battery, and blood samples. The middle column pictures from top to bottom are exercise, physical activity tracker, and home sleep headband.

We conducted a pre–post-interventional study. Adults aged 50 to 75 years old were recruited from the greater Boston area through online advertisements, flyers, and outpatient clinics at the Massachusetts General Hospital. Participants were included if physically inactive (average time spent exercising was less than 60 min per week in the past 6 months) and if they received clearance from their primary care physician to participate in the 12-week moderate-intensity exercise program. A pre-screening survey was used to select eligible participants with the following exclusion criteria: (1) a history of neurological illness (e.g., poorly controlled epilepsy with >1 seizure per month in the last 6 months, stroke with residual motor language deficits, multiple sclerosis, PD, dementia, head trauma in the past 6 months with a loss of consciousness >30 min, cerebral palsy, brain tumor, normal-pressure hydrocephalus, or Huntington’s disease; (2) a history of untreated or poorly controlled neuropsychiatric illness; (3) diagnosed with moderate or severe sleep apnea (apnea–hypopnea index ≥ 15/hour of sleep) or using a continuous positive airway pressure machine (CPAP); (4) HIV infection; (5) a history of falling within the past 6 months; (6) the inability to safely exercise (e.g., coronary heart disease, heart failure, osteoarthritis, or chronic fatigue syndrome) or the inability to perform any of the tests, e.g., failure to complete cardiopulmonary exercise testing (CPET) due to the development of cardiac symptoms during testing, or a lack of English proficiency, which would limit cognitive testing; and (7) the inability to complete the daily workout schedule. Written informed consent was obtained prior to participation in the study, which was approved by the Mass General Brigham Review Board (National Clinical Trial: NCT04210882). On the day of consent, participants completed CPET, cognitive testing, and a blood draw. Participants returned to the hospital within one week to undergo in-lab diagnostic polysomnography (PSG). These procedures were repeated at the end of the study. After the initial PSG, participants were instructed to begin a program of moderate-intensity exercise. Details of the exercise program are described below. The study design is shown in Figure 1.

Power analysis suggested that a minimum of 34 subjects (80% power, 0.5 mean of paired differences, 1.0 standard deviation of differences [30]) would be needed to evaluate whether a 12-week moderate-intensity exercise program can improve BAI and cognitive scores. We proposed the recruitment of 35 subjects to complete all assessments over a 1-year period to establish adequate power and to minimize Type II errors.

### 2.2. Cardiopulmonary Exercise Testing

Aerobic exercise capacity (maximal oxygen consumption, VO_2_max), resting heart rate, and cardiac-event risk were measured at the Brigham and Women’s Hospital CPET laboratory by a clinical exercise physiologist. Prior to the visit, participants were instructed to refrain from smoking, eating, and drinking caffeinated or alcoholic beverages for an 8–12 h period overnight. During the exam, participants’ VO_2_max was determined by the following ramp cycle protocol: cycle ergometer (Lode Corival, Lode BV, Groningen, The Netherlands) intensity was set to 10–25 watts/minute (depending on the subject’s capacity) at the speed of 60 rpms until exhaustion or clinical concerns were voiced. Otherwise, termination criteria are listed below as follows: (1) participant heart rate reached the maximal value of (220 − age); (2) oxygen intake reached a plateau despite an increase in work rate; and (3) participants who indicated exhaustion or the BORG scale was equal to or larger than 17. Respiratory measurements and electrocardiogram (ECG) were recorded by the Ultima CPX system (Medgraphics, Saint Paul, MN, USA). For two participants who refused to complete the final assessment of their VO_2_max due to COVID-19 concerns, VO_2_max was estimated by a 1-mile walk test on an outdoor track. Briefly, participants were instructed to walk as quickly as possible while avoiding jogging or running. Post-walking heart rate, walk time, body weight, age, and sex were used to calculate VO_2_max as described in a previous study [31]. In the context of the pandemic, where many participants expressed reluctance to visit hospitals due to concerns regarding viral transmission, utilization of the 1-mile walk test as a substitute for standard measurements was felt to be justified. A previous study validated that the correlation of VO_2_max measurements between the 1-mile walk test and graded treadmill testing was 0.8 among middle-aged and older adults, suggesting that the 1-mile walk test provides a reasonable estimate of VO_2_max [32].

### 2.3. Cognitive Testing

The National Institutes of Health (NIH) Toolbox Cognition Battery was administered in a quiet and private environment by a trained study staff member [33]. This battery is one of the core domains of the NIH Toolbox for Assessment of Neurological and Behavioral Function and consists of seven instruments. Fluid cognition is a measure of five of these instruments Dimensional Change Card Sort (DCCS), flanker inhibitory control and attention (FICA), list sorting working memory (LSWM), pattern comparison processing speed (PCPS), and Picture Sequence Memory (PSM), whereas crystallized cognition is a composite of two of these instruments (Picture Vocabulary Test (PVT) and Oral Reading Recognition (ORR)). Total cognition is a composite of fluid and crystallized scores. Absolute scores for each of the seven tests and the three composite scores were used for analyses (all non-age adjusted). Further explanation of the individual tests and scoring procedures is available in the Supplementary Materials.

### 2.4. Plasma Biomarkers Level Assessment

On the morning of the CPET appointment, overnight fasting blood samples were drawn between 8 AM and 11 AM by a phlebotomist at the Mass General Brigham Center for Clinical Investigation. Blood was centrifuged at 2000× *g* for 15 min. Plasma was aliquoted and stored in a −80° freezer for protein analysis. Levels of cytokines were measured using the established electrochemi-luminescence-based multi-array method through the Quickplex SQ 120 system (by the Meso Scale Diagnostics LLC, Rockville, MD, USA) using previously reported methods [34,35,36]. In brief, the system utilizes a 96-well-based high-throughput readout. Human proinflammatory multi-plex kits were utilized, which enabled the detection of 8 cytokines in our samples, including INF-γ, TNF-α, IL-2, IL-4, IL-6, IL-8, IL-10, and IL-13. Procedures for measuring cytokines levels followed manufacturer protocols. In brief, our samples and standard proteins provided by the manufacturer were prepared and incubated at 4 °C overnight. Then, the mixed solutions were placed on a shaker for 2 h, followed by washing and then incubation with detection of antibodies for another 2 h. Next, the electrochemi-luminescence signals were captured through the SQ 120 system. Finally, protein concentrations (pg/mL) were calculated using manufacturer-provided standard concentrations.

### 2.5. Diagnostic Polysomnography (PSG)

PSG was performed at the American Academy of Sleep Medicine (AASM)-Accredited Massachusetts General Hospital Sleep Disorders Unit. During the COVID-19 pandemic, all participants were cleared with PCR COVID-19 testing 72 h prior to the study. A minimum of six hours of overnight sleep was monitored using conventional in-lab polysomnography (Compumedics, Charlotte, NC, USA) with a 250 Hz sampling rate and a 0.3–35 Hz bandpass filter. EEG data were recorded from frontal (F3 and F4), central (C3 and C4), and occipital (O1 and O2) electrodes, and then referenced to contralateral mastoid electrodes (M2 and M1). Electrooculogram (EOG), electromyogram (EMG), ECG, pulse oximetry, respiration, and nasal flowmeter were used to assess sleep apnea and leg movements. Sleep was staged as non-rapid eye movement (NREM) stages 1 to 3, R (REM), or awake (W) stages in consecutive 30 s epochs following AASM criteria by Registered Polysomnographic Technologists [37]. The final sleep report was reviewed by a physician with board certification in Sleep Medicine. Two participants who completed the initial assessment and exercise program declined to complete the final PSG assessment because of COVID-19 concerns. Their final sleep EEGs were recorded by Dreem 2, which is comparable to PSG to acquire physiological sleep signals [38].

### 2.6. 12-Week Exercise Regimen

Participants completed a 12-week exercise regimen designed as follows: maintenance of 50–75% maximal heart rate (HRmax = 220 − age) intensity for 30 min per day, 5 days per week. Participants began with 15 min/day × 3 days in week 1, 20 min/day × 4 days in week 2, and then 30 min/day × 5 days over weeks 3–12. If they were unable to exercise for 30 min continuously, participants were allowed to split the 30 min of exercise into sessions of 10–15 min with 5 min rest intervals. For feasibility, the type and timing of exercise were not restricted; however, participants were encouraged to exercise around the same time each day. Participants were given a physical activity tracker (Fitbit, San Francisco, CA, USA) to monitor their exercising heart rate to ensure that exercise time and intensity met the requirements. Additionally, participants were instructed to log each physical activity session. High-intensity interval training (HIIT) and strength training were not allowed. According to the logs, 84% of participants’ exercise type was jogging or fast walking, 8% was elliptical running, and the remainder was cycling. The study team met in person or via teleconference (during the COVID-19 pandemic) bi-weekly with participants to answer questions and to verify that exercise duration adhered to the stipulated requirements.

### 2.7. Home Data Collection

Participants were asked to wear the Fitbit 24 × 7 and to sync the device every 5 days to upload the data to the cloud, allowing the study team to check adherence to the exercise program. Additionally, overnight sleep EEG was recorded using a home sleep headband (Dreem 2, France, or Prodigy, Canada) 2 nights per week throughout the 12-week exercise program. The Dreem 2 headband records via five EEG dry electrodes (Fpz, F7, F8, O1, and O2) at a 250 Hz sampling rate with a 0.4–35 Hz bandpass filter. The Prodigy sleep monitor records via six snap forehead electrodes (left/right frontal EEG sensors, left/right EOG sensors, mastoid, and chin sensors) at a 120 Hz sampling rate with a 0.33–35 Hz bandpass filter [39]. Each subject used the same device throughout the study for consistency. Heart rate and EEG data were checked by the study team weekly to ensure adherence.

### 2.8. Exercising Time and Sleeping Heart Rate

Continuous heart rate was tracked using a Fitbit via an optical sensor every 10 to 15 s. We set three timepoints to determine the time of exercise: T1 (peak heart rate timepoint), T2 (45 min before T1), and T3 (45 min after T1). Periods of exercise sessions were defined as compliant with the study protocol when the heart rate during T2 and T3 was ≥50–75% HRmax for at least 10 min. The sum of all such sessions was defined as the total exercise time. In the absence of Fitbit data, the exercise log was used. Fitbit automatically records sleep and wake timepoints. Sleeping heart rate was defined as the average heart rate across the entire night of sleep. We empirically identified periods during sleep when the heart rate was lower than the 20th percentile as representing stage N3, and periods with heart rate greater than the 80th percentile as REM.

### 2.9. EEG Preprocessing and Artifact Removal

For both in-lab PSG and at-home sleep measurements, EEG signals were notch-filtered at 60 Hz to reduce line noise and bandpass filtered from 0.5 Hz to 20 Hz to reduce myogenic artifact. The signals were then segmented into 30 s epochs. Epochs with artifacts were excluded in two steps. In the first step, we removed “definite” artifacts by excluding epochs with a maximum absolute amplitude larger than 500 µV or a flat (standard deviation < 1 µV) amplitude that lasted 2 s or longer. In the second step, for the remaining epochs, we trained a linear discriminant analysis (LDA) classifier to classify each epoch into artifact vs. not artifact. We used the total power and 2nd-order difference (for abrupt non-physiological changes; see the Supplementary Materials) of the spectrum as inputs to the LDA classifier. To train the classifier, we manually labeled each epoch in randomly selected EEGs (two channels: F7-O1, F8-O2) with different ratios of definite artifact per epoch across the night: 10 EEGs with a ratio of 25–50%; 10 EEGs with a ratio > 50%. After training, the LDA classifier labeled each epoch as artifact or not artifact. By comparing these with manually assigned labels, we derived a receiver operating characteristic (ROC) curve (Figure S1). We used this ROC curve to find a threshold that achieved a false negative rate (FNR) of 10%, as visual analysis suggested that this cutoff produced an acceptable tradeoff between retaining high-quality signals and rejecting artifactual epochs. The EEG artifact removal model mentioned above was implemented using Python 3.7 (Python Software Foundation, Wilmington, DE, USA) and the MNE package (https://mne.tools/stable/index.html on 1 May 2020). Please check Supplemental Methods S1 for details regarding the artificial removal of EEG.

### 2.10. Brain Age Computation and Spindle Analysis

The BAI was computed as described previously, with minor changes [28]. Briefly, BAI was calculated using a machine learning model with overnight sleep EEG features. For each 30 s epoch, we extracted 96 features from both time and frequency domains, including line length (signal complexity) and signal kurtosis (extent of extreme values); max, min, mean, and standard deviation of relative delta, theta, alpha band powers, and delta-to-theta, delta-to-alpha, and theta-to-alpha power ratio computed across 2 s sub-epochs; and kurtosis of spectral power in delta, theta, alpha, and sigma bands. We averaged these features within each of the five sleep stages separately (REM, N1, N2, N3, and Wake) over time, and concatenated them, to finally arrive at 96 × 5 = 480 features to represent physiologic information in a night of sleep. Here, we extracted 96 features, whereas our previous study used 102 features. The 6 fewer features are the sample entropy of the EEG from each of the 6 electrodes. Further examination showed that these features had zero coefficients in the original BAI model. Therefore, we dropped them. Note that the 96 features are for computing BAI from overnight in-lab PSG at the beginning and the end of the study, which includes 6 EEG electrodes. When computing BAI from at-home sleep EEGs (Dreem and Prodigy), we used the available 2 frontal EEG electrodes. Hence, the feature number was 96/3 = 32. The brain age model for frontal EEG only was trained using the same training dataset from the original brain age model, while limiting features to the 2 frontal EEG electrodes only. Sleep EEG data with an artifact ratio < 0.5 were computed for the BAI. In addition, sleep spindles, delta or slow oscillations, and their coupling during N2 sleep were detected and their summary features were quantified using Luna software V0.27 (27 September 2022) [40]. In detail, the signal is first bandpass filtered at 0.2–4.5 Hz using Luna, and then SOs are detected using zero-crossing rules. The signal for detecting SO is the raw signal from the device; there is no high-pass filtering performed. Spindle features included spindle density, average peak frequency, average amplitude, and average duration. The delta or slow oscillation features include their density, average amplitude, average duration, positive-to-negative slope, and negative-to-positive peak. The coupling features included magnitude of coupling, number of spindles overlapping a detected delta or slow oscillation, and average delta or slow oscillation phase at the spindle peak.

### 2.11. Sleep Metrics Analysis

Sleep stages and conventional sleep metrics were analyzed for in-lab PSG measurements and home sleep assessments. Sleep staging of in-lab PSGs was performed as described above. Prodigy headband data were staged using a previously described automated algorithm [41]. Dreem headband data were staged using the manufacturer’s automated system [38]. Additionally, conventional sleep metrics were calculated as follows:

- Sleep efficiency = TST/TIB × 100%;
- Awakening index = (# of transitions sleep to wake)/TIB;
- WASO = total wake time after sleep and before final awakening;

where TST = total sleep time; TIB = time in bed; WASO = wake after sleep onset.

### 2.12. Statistical Analysis

The change in various metrics from pre- to post-exercise, including BAI, sleep macro- and micro-architecture, cognitive scores, VO_2_max, and heart rate, were analyzed with paired t-tests. Effect size (ES, Cohen’s d) was calculated as the mean change of pre-exercise and post-exercise values divided by pooled standard deviation (no adjustment of covariates, as the study compared pre- and post-exercise values within the same subjects). Associations were measured using Pearson’s correlation coefficients [42]. Shapiro–Wilk tests were used to verify the normal distribution requirement of Pearson’s correlation analysis. A linear mixed effects model (LMEM) [43] was employed to examine the effects of age, gender, BAI, VO_2_max, heart rate, cognitive scores, and cytokines that model both pre-to-post within-person effects and cross-sectional effects. The mixed effect models used a random intercept and random slope. Benjamini and Hochberg False Discovery Rate (FDR) method to control the proportion of false discoveries when conducting multiple hypothesis tests. Data are presented as mean ± standard error (SE), mean difference (ME, post-pre data), and 95% confidence interval (CI), except where otherwise specified. Statistical significance was defined as *p* < 0.05. Statistical analyses were performed with Python 3.7 (Python Software Foundation, Wilmington, DE, USA), Rstudio 1.1.0 [44], and GraphPad Prism 8.0 (San Diego, CA, USA).

## 3. Results

### 3.1. Participant Characteristics

**Figure 2:**
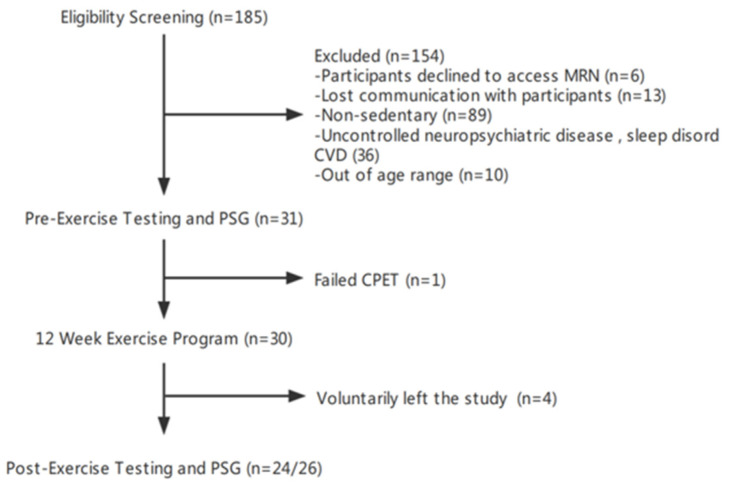
Enrollment flow chart. The present study flow was eligibility screening, pre-exercise testing, 12-week exercise regimen, and post-exercise testing.

Thirty-one eligible participants were enrolled between November 2019 and June 2021 (Figure 2). Five were subsequently excluded: one withdrew because of cardiovascular risk detected by CPET; two withdrew for personal reasons related to the COVID-19 pandemic; and two withdrew due to stated inability to exercise resources during the pandemic. Of the remaining twenty-six participants, two declined to complete the final in-lab PSG and CPET assessments due to COVID-19 concerns. Their final sleep EEG and VO_2_max were assessed outside of the hospital (see Section 2 above). Prior to reaching the pre-defined target sample size of 34, recruitment was discontinued due to unanticipated enrollment and budget challenges related to the COVID-19 pandemic. Thus, all analyses reported herein are based on the participants who completed the study (N = 26), except where otherwise specified.

Our final sample included 20 females and 6 males with an average age of 60 ± 7.37 years (mean ± standard deviation, SD). All subjects completed at least college-level education, averaging 17.19 ± 2.8 (mean ± SD) years of education. The average body mass index (BMI) was not significantly different between pre- and post-exercise time points. Average apnea–hypopnea indices were 4.05 ± 3.92 (mean ± SD) and 3.06 ± 3.94 (mean ± SD) at initial and final assessments. Participants exercised for approximately 47 ± 13 days (mean ± SD) over the 12-week period and 55 ± 9.8 min/day (mean ± SD), based on their heart rate data and exercise logs. Participant characteristics are presented in Table 1.

### 3.2. Physical Fitness

**Figure 3:**
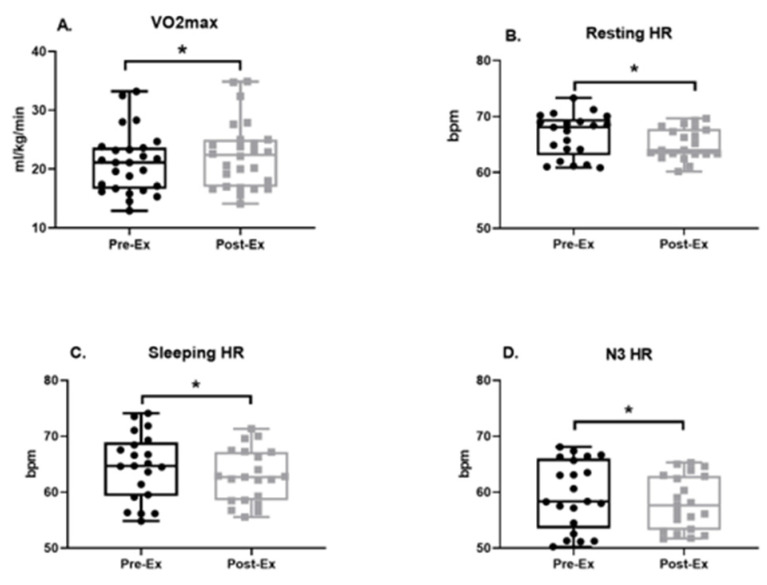
Comparison of baseline (Pre-Ex) and after 12 weeks of exercise (Post-Ex) cardio fitness data via measurement of maximum oxygen consumption (VO_2_max, (**A**)), resting heart rate (resting HR), sleeping HR, and N3 HR (**B**–**D**). N = 26, data are mean ± standard error. * is set as statistically significant, *p* < 0.05.

The 12-week aerobic exercise program improved measures of cardiovascular fitness, as measured by VO_2_max (Pre-Ex: 21.11 ± 1.03 [18.98, 23.23] mL/kg/min, Post-Ex: 22.39 ± 1.12 [20.09, 24.68] mL/kg/min; ME = 1.28, ES = 0.33, *p* = 0.0413; Figure 3A). Relative to baseline measurements, decreases in resting heart rate (Pre-Ex: 66.66 ± 0.83 [64.92, 68.4] bpm, Post-Ex: 65.13 ± 0.61 [63.87, 66.4] bpm, ME = −1.53, ES = −0.02, *p* = 0.011; Figure 3B), sleeping heart rate (Pre-Ex: 64.55 ± 1.28 [61.87, 67.23] bpm, Post-Ex: 62.93 ± 1.03 [60.78, 65.09] bpm; ME = −1.62, ES = −0.15, *p* = 0.028; Figure 3C), and heart rate in N3 sleep (Pre-Ex: 59.59 ± 1.33 bpm [56.82, 62.35], Post-Ex: 58.11 ± 1.05 [55.91, 60.30] bpm; ME = −1.48, ES = −0.14, *p* = 0.0314; Figure 3D) were also observed following the exercise program.

### 3.3. Cognition Performance Score

**Figure 4:**
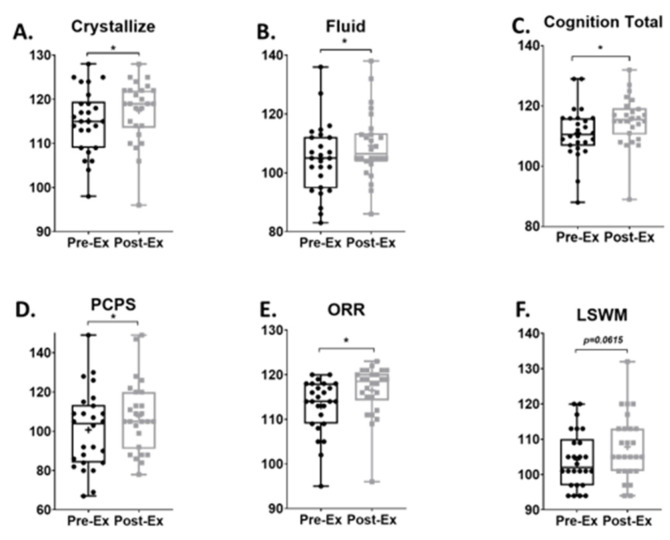
Comparison of baseline (Pre-Ex) and after 12 weeks of exercise (Post-Ex) cognitive function scores. Crystallize cognition score (**A**); fluid cognition score (**B**); total cognition score (**C**). Pattern comparison processing speed score (PCPS, (**D**)); oral reading recognition score (ORR, (**E**)); list sort working memory score (LSWM, (**F**)). N = 26, data are mean ± standard error. * is set as statistically significant, *p* < 0.05.

After the 12-week exercise program, cognitive performance was improved in multiple domains, including the composite domains of crystallized intelligence (Pre-Ex: 115.1 ± 1.4 [112.1, 118], Post-Ex: 117.4 ± 1.38 [114.5, 120.2]; MS = 2.3, ES = 0.32, *p* = 0.0008; Figure 4A), fluid intelligence (Pre-Ex: 104.8 ± 2.35 [99.98, 109.7], Post-Ex: 109.3 ± 2.23 [104.7, 113.9]; MS = 4.5, ES = 0.38, *p* = 0.0058; Figure 4B), and total cognition (Pre-Ex: 111.1 ± 1.70 [107.6, 114.6], Post-Ex: 115.2 ± 1.60 [111.9, 118.5]; MS = 4.1, ES = 0.49, *p* = 0.0001; Figure 4C). Additionally, improvements in the subdomains of processing speed, as measured by pattern comparison processing speed (Pre-Ex: 100.7 ± 3.92 [92.64, 108.8], Post-Ex: 107.6 ± 3.52 [100.4, 114.9]; MS = 6.9, ES = 0.36, *p* = 0.036; Figure 4D), and language skills, as measured by oral reading recognition (Pre-Ex: 113 ± 1.19 [110.5, 115.5], Post-Ex: 116.5 ± 1.13 [114.2, 118.8]; MS = 3.5, ES = 0.59, *p* < 0.0001; Figure 4E), were observed. Improvement on the list sort working memory test was marginally significant (Pre-Ex: 104.2 ± 1.56 [101.0, 107.4], Post-Ex: 107.8 ± 9.11 [104.2, 111.5]; MS = 3.6, ES = 0.42, *p* = 0.0615; Figure 4F).

### 3.4. Plasma Biomarkers

**Figure 5:**
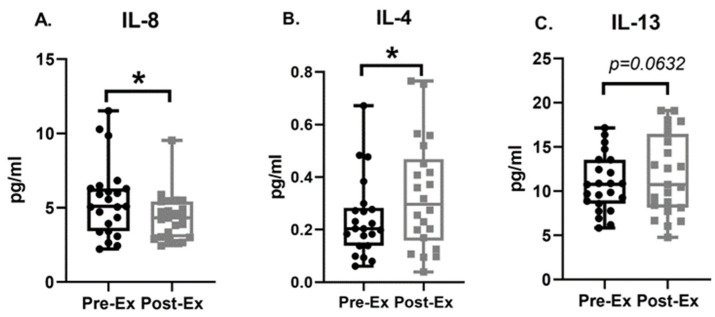
Comparison of baseline (Pre-Ex) and after 12 weeks of exercise (Post-Ex) plasma cytokines level, interleukin-8 (IL-8, (**A**)), IL-4 (**B**), and IL-13 (**C**). N = 24, data are mean ± standard error. * is set as statistically significant, *p* < 0.05.

Compared to baseline levels of plasma cytokines, the post-exercise IL-8 level was significantly decreased (Pre-Ex: 5.50 ± 0.51 [4.45, 6.55] pg/mL, Post-Ex: 4.34 ± 0.33 [3.66, 5.01] pg/mL; MS = −1.16, ES = −0.57, *p* = 0.0022, Figure 5A). After 12 weeks of moderate-intensity exercise, plasma IL-4 (Pre-Ex: 0.24 ± 0.03 [0.18, 0.31] pg/mL, Post-Ex: 0.33 ± 0.04 [0.24, 0.42] pg/mL; MS = 0.09, ES = 0.49, *p* = 0.0330, Figure 5B) was significantly elevated compared to baseline. Peripheral IL-13 level (Pre-Ex: 10.84 ± 0.66 [9.46, 12.21] pg/mL, Post-Ex: 11.90 ± 0.95 [9.93, 13.87] pg/mL; MS = 1.06, ES = 0.27, *p* = 0.0632, Figure 5C) borderline significantly increased after the exercise regimen.

### 3.5. BAI and Sleep Micro-Architecture

**Figure 6:**
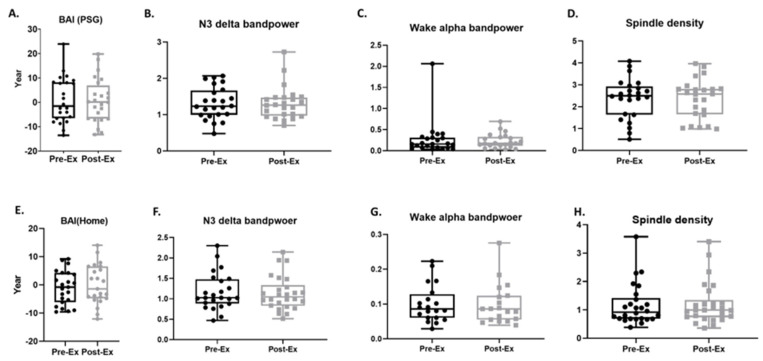
Comparison of baseline (Pre-Ex) and after 12 weeks of exercise (Post-Ex) brain age index and sleeping micro-architectures. Polysomnography (PSG) data, N = 24 (**A**–**D**) and home device data, N = 26, (**E**–**H**). Data are mean ± standard error.

No differences were seen in BAI measured through in-lab PSG comparing baseline and post-exercise timepoints (Pre-Ex: 0.87 ± 1.89 [−3.04, 4.78] years, Post-Ex: 0.69 ± 1.83 [−3.09, 4.47] years; MS = −0.18, *p* = 0.9026; Figure 6A). Additionally, we did not observe significant changes in sleep micro-architectural features, including delta band power during N3 sleep (Pre-Ex: 1.31 ± 0.09 [1.13, 1.50] dB, Post-Ex: 1.31 ± 0.09 [1.21, 1.5] dB; MS = −0.002, *p* = 0.9734; Figure 6B) or alpha band power in the awake state (Pre-Ex: 0.27 ± 0.09 [0.09 0.45] dB, Post-Ex: 0.23 ± 0.04 [0.15, 0.3] dB; MS = −0.04, *p* = 0.5238; Figure 6C). Likewise, no change was observed in spindle density (Pre-Ex: 2.37 ± 0.19 [1.98, 2.76], Post-Ex: 2.35 ± 0.18 [1.97, 2.73]; MS = −0.02, *p* = 0.8420; Figure 6D). After artifact detection and removal, 64% of home sleep headband data exhibited sufficient quality (artifact ratio < 0.5) to compute BAIs. As with in-lab PSG findings, no significant differences were seen in BAI measured by home sleep EEG when comparing baseline to post-exercise data (Pre-Ex: −0.95 ± 1.24 [−3.52, 1.62] years, Post-Ex: 0.13 ± 1.46 [−2.89, 3.15] years; MS = 1.08, *p* = 0.3652; Figure 6E). Similarly, we found no significant changes in delta band power during N3 sleep (Pre-Ex: 1.17 ± 0.09 [0.98, 1.36] dB, Post-Ex: 1.13 ± 0.08 [0.96, 1.30] dB; MS = −0.04, *p* = 0.6447; Figure 6F), alpha band power in the awake state (Pre-Ex: 0.1 ± 0.05 [0.07, 0.16] dB, Post-Ex: 0.09 ± 0.06 [0.07, 0.13] dB; MS < 0.01, *p* = 0.6849; Figure 6G), or spindle density (Pre-Ex: 1.17 ± 0.14 [0.88, 1.46], Post-Ex: 1.21 ± 0.15 [0.91, 1.51]; MS = 0.04, *p* = 0.4585; Figure 6H).

### 3.6. Sleep Macro-Architecture

**Figure 7:**
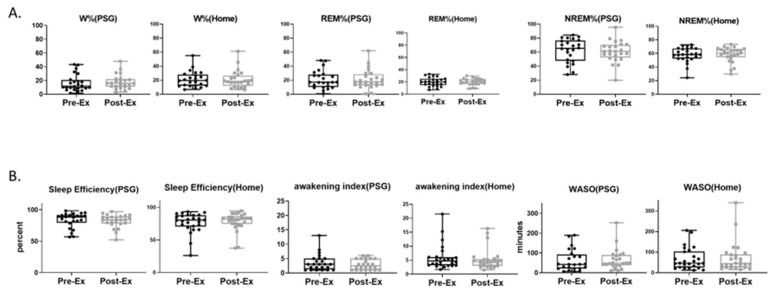
Comparison of baseline (Pre-Ex) and after 12 weeks of exercise (Post-Ex) conventional metrics of sleep quality. Percents of sleep stage: wake, rapid eye movement, non-rapid eye movement (W, REM, and NREM, (**A**)),. Sleep fragmentation data: sleep efficiency (**B**), awakening index (**B**), and wake after sleep onset (WASO, (**B**)). Data are mean ± standard error. PSG data (N = 24; home device data (N = 24).

We analyzed conventional metrics of sleep quality based on sleep macro-architecture from in-lab PSG and home sleep device data, to determine whether the 12-week exercise regimen was able to improve sleep quality. We found no significant differences in sleep stage percentages in the pre–vs.–post-exercise in-lab PSG data (Figure 7A) for wake (Pre-Ex: 16.63 ± 2.46 [11.54, 21.72]%, Post-Ex: 17.83 ± 2.12 [13.45, 22.21]%; MS = 1.2, *p* = 0.6498), REM (Pre-Ex: 20.88 ± 2.62 [15.45, 26.30]%, Post-Ex: 21.31 ± 2.80 [15.50, 27.12]%; MS = 0.43, *p* = 0.8107), and NREM states (Pre-Ex: 62.49 ± 3.39 [55.47,69.52]%, Post-Ex: 60.86 ± 3.06 [54.52, 67.20]%; MS = −1.63, *p* = 0.5982). Similarly, we found no significant differences in stage percentages after the 12-week exercise regimen compared to baseline measurement for sleep measured with home EEG (Figure 7A) for wake (Pre-Ex: 21.27 ± 2.3 [16.50, 26.03]%, Post-Ex: 21.18 ± 2.57 [15.87, 26.50]%; MS = −0.09, *p* = 0.9659), REM (Pre-Ex: 19.95 ± 1.45 [16.95, 22.94]%, Post-Ex: 19.94 ± 1.14 [17.58, 22.29]%; MS = −0.01, *p* = 0.9932), and NREM (Pre-Ex: 57.99 ± 2.15 [53.57, 62.42]%, Post-Ex: 58.53 ± 2.15 [54.10, 62.97]%; MS = 0.54, *p* = 0.7493).

We also found no significant exercise effects on sleep quality metrics derived from sleep macro-architecture measured with in-lab PSG (Figure 7B), including sleep efficiency (Pre-Ex: 83.37 ± 2.46 [78.28, 88.46]%, Post-Ex: 82.17 ± 2.12 [77.79, 86.55]%; MS = −1.2, *p* = 0.6498), awakening index (Pre-Ex: 3.58 ± 0.58 [2.39, 4.78], Post-Ex: 2.91 ± 0.36 [2.17, 3.67]; MS = −0.67, *p* = 0.2592), and wake after sleep onset (WASO) (Pre-Ex: 61.52 ± 11.11 [38.55, 84.50] mins, Post-Ex: 66.58 ± 10.77 [44.31, 88.86] mins; MS = 5.06, *p* = 0.7250). Similarly, no exercise effects were found in home sleep data (Figure 7B) for sleep efficiency (Pre-Ex: 76.8 ± 2.99 [70.65, 82.95]%, Post-Ex: 78.27 ± 2.72 [72.65, 83.89]%; MS = 1.47, *p* = 0.3697), awakening index (Pre-Ex: 5.96 ± 0.86 [4.18, 7.74], Post-Ex: 5.26 ± 0.72 [3.77, 6.76]; MS = −0.70, *p* = 0.2160), and WASO (Pre-Ex: 67.27 ± 10.39 [45.87, 88.67] mins, Post-Ex: 71.5 ± 14.21 [42.24, 100.8] mins; MS = 4.23, *p* = 0.5903).

### 3.7. Associations of the Study Outcomes

We further analyzed the relationship between the study outcomes by Pearson’s correlation (Table 2). The improvement of VO_2_max was positively (*p* = 0.0050) related to the enhancement of cognition performance but not related to the change in BAI (*p* = 0.8914). Elevated BAI was related to reduced NREM (*p* = 0.0035) sleep and more fragmented sleep as measured by wake stage percent (*p* = 0.0099), sleep efficiency (*p* = 0.0099), and WASO (*p* = 0.0252). However, we did not observe any statistically significant relations between δVO_2_max and δN3 (*p* = 0.6416). In addition, the increment of BAI was associated with lower levels of plasma IL-4 (*p* = 0.0240) and IL-13 (*p* = 0.0023). The improvement of cognitive executive function measured by dimension card change sort was positively related to increased N2 (*p* = 0.0150) and NREM (0.0370) sleep percentages. Additionally, we found higher levels of IL-13 and IL-4 and lower levels of IL-8 were significantly related to better sleep quality as measured by sleep efficiency (*p* = 0.0262), N3 percentage (*p* = 0.0062), NREM percentage (*p* = 0.0081), awakening index (*p* = 0.0341), and delta band power during N3 sleep (*p* = 0.0435). Higher levels of IL-4 were also significantly associated with the improvement of cognition performance measured by processing speed and language ability.

Results of the linear mixed effects modeling to analyze relations between BAI, VO_2_max, heart rate, cognitions, and plasma cytokines, adjusted for age and sex, are shown in Table 3. The results were computed from the whole dataset including the five withdrawn participants who only completed baseline assessments. Resting heart rate was negatively associated with VO_2_max (*p* = 0.03). Moreover, BAI predicted from PSG data was positively related to plasma IL-8 (*p* = 0.035), and inversely related to fluid intelligence (*p* = 0.009), processing speed (*p* = 0.002), IL-4 (*p* = 0.009), IL-13 (*p* < 0.0001), and potentially memory performance (*p* = 0.103). Additionally, we found a trend between PSG-derived BAI and resting heart rate, a predictor of VO_2_max (*p* = 0.087). However, BAI calculated from PSG data was not correlated directly with VO_2_max (*p* = 0.564). We did not observe any significant relationships between BAI calculated from home EEG data and VO_2_max, cognitive performance measures, heart rate, or cytokine levels.

## 4. Discussion

This interventional clinical trial evaluated the feasibility of using home monitoring devices and BAI to track brain health in physically inactive middle-aged and older adults over a 12-week moderate-intensity exercise program. Compared to previous studies that relied on subjective sleep reports or coarse measures of sleep, our study collected abundant physiological data in both in-lab and home settings. Our results suggest that (1) plasma cytokines, cognitive performance, and physical fitness were improved in physically inactive middle-aged and older adults after a 12-week moderate-intensity aerobic exercise regimen; (2) BAI, as currently computed, was not sensitive enough to detect neurophysiologic correlates of these improvements in cognition for this type of population after this level of improved physical fitness; (3) the improvement of cognitive performance was associated with improvements in aerobic fitness and higher circulating IL-4/13, although not with BAI; and (4) lower BAI was associated with less fragmented sleep and higher levels of neuroprotective cytokines (IL-4/13). Additionally, the present study is a feasible and exploratory single-arm trial that evaluated the combination of home devices and BAI to track brain and sleep health in middle-aged and older adults long term. Our physiological assessment suggests that moderate-intensity aerobic exercise may improve brain health and sleep quality in previously physically inactive middle-aged and older adults.

### 4.1. Improvements in Cognitive Performance

We found that the 12-week, 150 min per-week moderate-intensity exercise program produced measurable improvements in fluid intelligence, crystallized intelligence, and total cognitive performance. Specifically, processing speed and oral reading recognition skills were moderately improved, and improvements in working memory were marginally significant. Our results agree with previous reports showing moderate improvements in processing speed (~7%) following aerobic exercise in older adults [45,46]. Moreover, our data agree with a recent study that 12-week strength training improves fluid cognition in older adults [47]. In contrast to processing speed, previous studies show mixed results regarding working memory improvement following aerobic exercise [45,48,49], likely due to different sample demographics and assessment methods for working memory. However, one meta-analysis found that healthy participants exhibit improvements in working memory following exercise, which is in line with our findings [49]. Further, to determine whether the improvement of cognitive performance is related to learning or practice effects, we compared the effect size of our cognition performance with a previously published study [33]. We found that the current 12-week exercise program produced higher effect sizes on cognition scores compared to the 7- to 21-day test–retest effect. Additionally, the 12-week interval is long enough to minimize the test–retest practice effect on cognition function according to the previous literature [50]. Therefore, we believe the improvements seen in cognition performance result from the 12-week aerobic exercise regimen.

There may be multiple mechanisms by which a moderate-intensity exercise regimen improves cognitive performance, including the following: (1) improved cerebrovascular function [51,52]; (2) increase in hippocampal volume [53] and reduction in the burden of white matter lesions [54]; and (3) regulation of peripheral cytokines and neurotrophic factors, such as Brain-derived Neurotrophic Factor (BDNF) [55,56], IL-6, cathepsin B, irisin [57], and IGF-1 [58]. Here, we found that several plasma cytokines, IL-4, IL-8, and IL-13, were altered after the exercise regimen. Importantly, we further observed that the improvement of processing speed and oral reading recognition skills were related to the increment of IL-4. Our results reinforce the key functions of anti-inflammatory cytokines IL-4/13, which have been shown to exert neuroprotective effects on activated microglia to protect neurons from injury and hippocampal volume loss [59,60,61]. Moreover, higher levels of IL-4 in both animal and human trials are associated with better cognitive performance [62,63], while increased circulating proinflammatory IL-8 is associated with lower memory and processing speed [64]. The potential principals were IL-4 and receptor-mediated signaling is responsible for neuron growth and survival and promotes other neuron growth factors expression such as nerve growth factor (NGF). The observation of reduced circulating IL-8 over the 12-week exercise program is consistent with the finding of a different exercise regimen, which showed a rapid decrease in IL-8 at 30 min after exercise [65]. Additionally, IL-8 is strongly associated with oxidative stress [66,67], which is related to impaired cognition function [68]. Thus, our data suggest that one mechanism for the observed therapeutic effects of exercise on brain cognitive health may be improvements in circulating plasma cytokines. It might also be of interest in the future to measure the effects of physical exercise on biological aging using a DNA methylation epigenetic clock in parallel with the EEG-based BAI [69]. The findings of our current study warrant future research to further characterize the mechanisms by which exercise improves cognitive functions.

### 4.2. Sleep and Sleep EEG-Based Brain Age Index

When evaluating sleep metrics, we did not observe changes between baseline and post-exercise training evaluations. Similar to previous work, the effects of exercise training on sleep macro- or micro-architectures remain uncertain, especially in people without sleep complaints [70]. For example, acute exercise training has been reported in some studies to increase delta band power [19,71] and N3 sleep duration [72]. However, another randomized study concluded that a 12-month moderate-intensity exercise program improved several objective measures of sleep but did not change measures of N3 sleep [14]. Unfortunately, we did not observe any changes in BAI over the 12-week study period. Multiple factors (e.g., small sample size, exercise intensity, duration, the time before sleep when exercise was completed, night-to-night variability, and individual differences in participants) could potentially explain these inconsistencies. Although the exercise regimen did not appear to reduce BAI, we did find that younger brain age (lower BAI) was associated with more N2 and N3 sleep and less fragmented sleep, which we interpret as supporting BAI as an indicator of brain health over longer time periods. Additionally, we observed that higher plasma IL-13 is significantly associated with increased sleep efficiency and increased N3 sleep as a percentage of total sleep, while higher IL-8 levels were related to more fragmented sleep. Our findings are in agreement with previous work that circulating IL-8 is significantly increased in sleep-disordered patients compared to healthy controls [64]. Moreover, the higher levels of the neuroprotective cytokines IL-4/13 were significantly associated with younger brain age, which is the first time an association has been shown between circulating biomarkers and BAI. Furthermore, we discovered that circulating IL-4 is positively correlated with N3 sleep power. Recently, there has been increasing interest in augmenting N3 sleep to improve cognition, e.g., through transcranial electrical stimulation [73,74] and controlled acoustic stimuli [75]. N3 sleep and increased slow wave power during sleep are associated with several desirable physiological effects, including enhanced glymphatic flow [76,77], stable breathing, and vagal dominance of heart rate variability [78]. Along with the relationship between sleep microarchitecture and cognitive health, we observed that better brain executive function is associated with more NREM sleep, which is similar to previous findings [79].

It is noteworthy that this study was completed during the COVID-19 pandemic. This global pandemic is strongly associated with widespread heightening of anxiety and depression levels [80,81], significant changes in lifestyle (increase in daily sedentary time and nap time) [82], and increased disturbances in sleep patterns and quality [83,84]. In the present study, the changes in sleeping heart rate and improvements in exercise performance were modest. It is possible that a more vigorous and prolonged program may show changes not evident in our study. Individual differences in brain macro- and micro-architecture during sleep may also be important, with exercise providing greater benefit in those with reduced baseline slow wave power.

### 4.3. Improvements in Aerobic Fitness

As hypothesized, the 12-week moderate-intensity aerobic exercise program increased VO_2_max and decreased resting and sleeping heart rate. Notably, our study included two participants whose final VO_2_max values were estimated using the 1-mile walk method, highlighting a gap in directly comparing predictive VO_2_max with CPET-VO_2_max. While the 1-mile walk test is a validated indirect measure correlated with VO_2_ peak, we acknowledge its limitations in replacing CPET for VO_2_max determination. CPET remains the gold standard, ensuring a more accurate and comprehensive assessment of VO_2_max. Concerns arise regarding the potentially limited responsiveness of specific individuals to low-intensity endurance training in terms of VO_2_max improvement, as indicated by a meta-analysis [85]. It is noteworthy that the participants included in this meta-analysis predominantly encompass young to middle-aged adults. Conversely, an alternate report suggests a substantial 16.3% enhancement in VO_2_max resulting from endurance training when compared to control groups [86]. We observed that heart rate during N3 sleep was reduced after the exercise regimen compared to baseline measurements. We found that improvements in VO_2_max were associated with improvements in cognitive performance and reduction of sleeping heart rate, but not with a change in BAI and sleep macro- and micro-architecture. Additionally, we did not find any associations between the improvement of VO_2_max and the change of expression of plasma cytokines either, which may suggest that exercise to improve cognition is via other biochemicals rather than the current 8 cytokines. Our data generally align with previous findings that describe increases in physical fitness and brain health following aerobic exercise [87,88,89] and a link between elevation of exercise capacity with improvements in cognitive health, suggesting that the current intensity and frequency of workouts promote brain health. However, we acknowledge that our criteria for N3 sleep heart rate were operational, based on a heart rate percentile (<20%). We conducted tests using various percentiles, including 10%, 15%, 20%, and 25%. Our explorations suggested that a cutoff of 20% best matched N3 based on the scored in-lab PSGs.

Finally, our mixed effects model result suggested a lack of association between BAI and VO_2_max, but did find significant correlations between BAI, fluid intelligence, processing speed, IL4, IL-13, and IL-8. Processing speed performance changes over the lifespan, peaking during early adulthood and decreasing thereafter [90,91]. Importantly, processing speed is among the most sensitive cognitive processes influenced by neurological illness [92,93,94,95]. In light of the findings of our investigation, our exploratory data indicate that the current exercise program appears to confer a degree of benefit in enhancing essential cognitive performance. Additionally, IL-4 and IL-13 are critical anti-inflammatory cytokines with effects on sleep [96] and cognitive performance [97].

## 5. Limitations and Future Directions

The lack of change pre- and post-exercise training seen for BAI and other sleep metrics might be due to suboptimal exercise regimen in both intensity and duration, ceiling–floor effects due to the relatively healthy samples [98], small sample size, and night-to-night variability of sleep measures, including BAI [99]. One major limitation is the limited data quality of home sleep EEG. We employed home sleep headbands to record multiple nights of sleep EEG to account for night-to-night variability of BAI calculations. However, after artifact detection, only 64% of home sleep EEG data had an artifact ratio of <0.5 and only 44% had an artifact ratio of <0.3. Thus, despite collecting abundant longitudinal home sleep EEG data, only a portion of this was suitable for analysis, limiting our ability to average over multiple close-together nights to detect more subtle changes in BAI. Another significant limitation was the absence of a placebo or control group, precluding us from unequivocally attributing the observed enhancements in cognitive function to the exercise intervention. Our rationale for adopting a within-subject before–vs.–after study design was to focus on changes in each participant’s parameters relative to their own baseline. While the inclusion of a control arm could have enhanced methodological rigor, it would have necessitated a larger sample size, a constraint precluded by budgetary considerations. Moreover, randomizing a subset of our limited number of subjects to refrain from exercise could have deterred interest in the study. Given these considerations and the exploratory nature of our research, we deemed the single-arm design to be best. In addition to these limitations, the lack of significant changes in sleep parameters and specific deep brain structures associated with sleep function following aerobic exercise also constitutes a limitation of the present study. Future research could aim to investigate the potential mechanisms underlying these observed effects, including exploring specific neural tracts or pathways modulated by physical activity, and, considering the duration, intensity, and type of exercise. Finally, most participants in this study are white college-educated females. A more diverse population should be included in future work.

We acknowledge that the utilization of a Fitbit for heart rate tracking in the current study presents another limitation. More dependable and precise measurements can be achieved through alternative methods such as chest straps or forehead photoplethysmography (PPG). However, we made a deliberate choice not to employ these alternatives due to practical considerations. Participants were tasked with wearing heart rate monitors continuously, 24/7, over the course of the 12-week study duration, a requirement we anticipated would be challenging to maintain with forehead PPG or a chest strap. Considering budget constraints, user-friendliness, public acceptance, and the feasibility of conducting future large-scale, long-term studies, we opted for a Fitbit over the Polar chest strap or PPG.

Another limitation relates to our measurement of oxygen capacity: our study did not perform VO_2_max verification testing. Implementation of the validation stage poses considerable challenges due to budget limitations, scheduling conflicts among participants, and the limited capacity of CPET laboratories in the hospital setting. It is important to note that no standardized protocol for executing the verification phase exists, necessitating further research efforts to establish a feasible protocol applicable to sedentary older adults [100].

It is notable that this small sample-size trial was a feasibility and exploratory study to determine whether BAI measured via home sleep monitoring and cognition were influenced by exercise. The commencement of this research occurred a few months prior to the emergence of the COVID-19 pandemic. This situation posed significant difficulties in enrolling a suitable cohort of participants. Unfortunately, owing to financial limitations and external factors, we were obliged to prematurely conclude the study before achieving our target enrollment number. To fully evaluate the effects of exercise on BAI, a larger sample size is essential. Future studies may also evaluate the effects of different intensities (low, medium, high) and duration exercise regimens on sleep and BAI. Our ultimate goal is to develop an affordable and accurate physiological measure for tracking brain health.

## 6. Conclusions

This exploratory study provided evidence that a 12-week, 150 min per-week moderate-intensity aerobic exercise regimen improves cognitive performance, particularly on cognitive processing speed in association with improved aerobic fitness and higher circulating neuroprotective cytokines (IL-4 and IL-13) among middle-aged and older previously physically inactive adults. Nonetheless, we did not detect significant enhancements in sleep macro- and micro-architectures, cognitive flexibility, attention, or the rapid acquisition and retention of new information.

## Figures and Tables

**Table 1:** Characteristics of Participants.

	Pre-Exercise	Post-Exercise
Age	60.11 ± 7.37	-
Sex (F, M)	26 (20/77%, 6/23%)	-
Race	1/3% (American Indian or Native Alaskan)	-
	3/12% (Asian)	
	1/3% (Black or African)	
	23/88% (White)	
YOE (SD)	17.19 ± 2.80	-
BMI (SD)	25.86 ± 4.16	25.83 ± 3.97
AHI	4.05 ± 3.92	3.06 ± 3.94
Exercise days	-	47 ± 13
Exercise time (minutes)	-	55 ± 9.8

Participant characteristics. YOE: years of education. BMI: body mass index. AHI: apnea–hypopnea index. Exercise time and days were counted when heart rate was 50–75% of max heart rate (220 − Age). One participant selected more than one race. Data are mean ± standard deviation.

**Table 2:** The associations between the change of aerobic fitness, cognition functions, sleep micro- and macro-architecture, and plasma cytokines.

Features		Pearson’s *r*	*p* Value
VO_2_max vs.	SHR	−0.47	0.0160
	FLD	0.65	0.0003
	cognition total	0.53	0.0050
	DCCS	0.62	0.0007
	FICA	0.49	0.0115
	LSWM	0.70	<0.0001
	PCPS	0.40	0.0441
	BAI	0.03	0.8914
BAI vs.	sleep efficiency	−0.53	0.0099
	WASO	0.47	0.0252
	Wake	0.53	0.0099
	N2	−0.44	0.0368
	N3	−0.42	0.0440
	NREM	−0.58	0.0035
	IL-13	−0.60	0.0023
	IL-4	−0.47	0.0240
DCCS vs.	N2	0.50	0.0150
	NREM	0.44	0.0370
IL-13 vs.	PVT	−0.47	0.0246
	sleep efficiency	0.46	0.0262
	Wake	−0.46	0.0262
	N3	0.55	0.0062
	NREM	0.54	0.0081
IL-4 vs.	PCPS	0.51	0.0134
	ORR	0.43	0.0412
	delta band power in N3	0.42	0.0435
IL-8 vs.	awakening index	0.44	0.0341

Pearson’s correlation to verify the associations between the change of aerobic fitness, cognition functions, sleep micro- and macro-architecture, and plasma cytokines. Maximal oxygen consumption: VO^2^max. SHR: sleeping heart rate. FLD: cognition fluid. DCCS: dimension change card sort. FICA: flanker inhibitory control and attention. LSWM: list sort working memory. PCPS: pattern comparison processing speed. ORR: oral reading recognition. PVT: picture vocabulary test. BAI: brain age index. WASO: wake after sleep onset. NREM (N): non-rapid eye movement. IL-4, 8, and 13: Interleukin-4, 8, and 13. N = 24.

**Table 3:** Mixed effects model.

Dependent var.	Independent var.	*p* Value	95%CI	Coefficient
VO_2_max	BAI	0.564	−0.14, 0.07	−0.03
	Resting HR	0.03	−0.29, −0.05	−0.15
BAI	VO_2_max	0.868	−0.66, 0.55	−0.05
	Resting HR	0.087	−0.5, 0.75	0.35
FLD	BAI	0.009	−0.5, −0.06	−0.28
PCPS		0.002	−0.32, −0.05	−0.18
LSWM		0.103	−0.4, 0.07	−0.17
IL−4		0.009	−22.83, −3.89	−13.36
IL−8		0.035	0.04, 2.17	1.1
IL−13		<0.0001	−1.64, −0.51	−1.08

Mixed linear regression model to calculate the associations of all pre- and post-exercise data. Var.: variable. VO_2_max: maximal oxygen consumption. BAI: brain age index. HR: heart rate. FLD: cognition fluid. PCPS: pattern comparison processing speed. LSWM: list sort working memory. IL-4, 8, and 13: Interleukin-4, 8, and 13. CI: confidence interval. N = 31.

## Data Availability

The original contributions presented in the study are included in the article/Supplementary Material, further inquiries can be directed to the corresponding authors.

## Supplementary Materials

The following supporting information can be downloaded at: https://www.mdpi.com/article/10.3390/life14070855/s1, Figure S1. The performance of linear discriminant analysis (LDA) to classify EEG artifact ratio, Table S1. Plasma biomarkers level, Table S2. (**A**) The associations between the changes of the physiological outcomes; (**B**). The associations between the changes of VO_2_max and physiological outcomes; (**C**). The associations between the changes of BAI and physiological outcomes; (**D**). The associations between the changes of IL-13 and physiological outcomes; (**E**). The associations between the changes of IL-4 and physiological outcomes; (**F**). The associations between the changes of IL-8 and physiological outcomes.
