# Supplementary material for: Effects of Aerobic Exercise on Brain Age and Health in Middle-Aged and Older Adults: A Single-Arm Pilot Clinical Trial
Source: Life (Basel). 2024 Jul 8;14(7):855. doi: 10.3390/life14070855 (PMC11278044; doi:10.3390/life14070855)
Supplement: Supplementary file 1 [file life-14-00855-s001.zip › life-2891550-supplementary.pdf]

Supplemental Methods

1. EEG signal pre-processing

EEG artifact removal: 2nd order difference in EEG spectrum computation
The 2nd order difference of spectrum is used to quantify the extent of single frequency non-physiological noise:
Step 1: convert the spectral power density (PSD, uV^2/Hz) to decibel (dB): $dB = 10 \log_{10} PSD$;
Step 2: standardize the spectrum to have standard deviation of 1 across frequency bins;
Step 3: get maximum absolute second order difference = $\max_{i=1,\ldots F-2} |(x_{i+2} - x_{i+1}) - (x_{i+1} - x_i)|$;
Step 4: take log to make its distribution closer to normal distribution.

Figure S1. The performance of linear discriminant analysis (LDA) to classify EEG artifact ratio

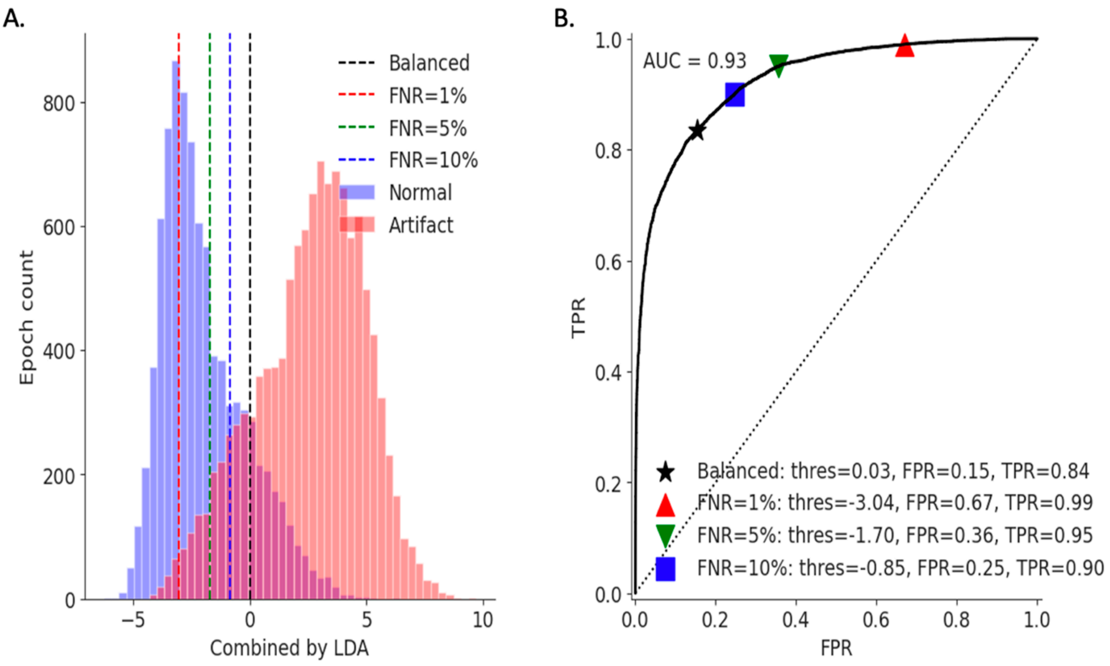

Figure S1. The performance of linear discriminant analysis (LDA) to classify EEG artifact ratio**.** The histogram of epochs labeled by human, where blue color is no artifact (normal), and red color is artifact. The x-axis is the linearly combined value of the total power and 2nd order difference (the two input features to LDA), indicating a score of how likely the epoch contains artifact. The y-axis is the count of epochs (Fig. S1A). The receiver operating characteristic curve (ROC), where the x-axis is the false positive rate, and the y-axis is the true positive rate. The vertical dashed lines are obtained by different thresholds for achieving a binary decision, obtained from the ROC. The perfect point is at the left upper corner and the random performance is indicated by the diagonal dashed line. On the ROC, we indicated four operating points, at

different false negative rates, and a balanced point where it is closest to the perfect point in terms of Euclidean distance (Fig. S1B).

2. NIH ToolBox Congnition Battery

In general, superior cognitive performance is associated with higher scores. Performance measures yield three types of scores: Age-Corrected Standard Scores, with a normative mean of 100 and a standard deviation (SD) of 15; Uncorrected Standard Scores (mean = 100, SD = 15); and Fully Corrected Scores, designed for neuropsychological use, adjusting for age, education, sex, and race/ethnicity influences. These Fully Corrected Scores adopt a T-Score metric with a normative mean of 50 and an SD of 10. In our current investigation, we portrayed all cognitive assessment data using the Uncorrected Standard Score. As delineated in the ToolBox Manual, this metric reflects the individual test-taker's performance in comparison to the extensive national normative sample established by the NIH Toolbox. It serves as a valuable indicator when gauging an individual's overall level of functioning, effectively sidestepping influences stemming from variables such as age, gender, or other demographic factors. Given the diversity in participant characteristics, we consider the Uncorrected Standard Score as the most suitable approach for mitigating the potential confounding effects of age and gender and other background factors.

Each individual test are described briefly based on official Toolbox Scoring and Interpretation Guide (https://www.nihtoolbox.org/app/uploads/2022/05/Toolbox_Scoring_and_Interpretation_Guide_for_iPad_v1.7-5.25.21.pdf)

2.1 Picture Vocabulary Test
The Picture Vocabulary Test assessment employs computerized adaptive testing, tailoring questions to each participant's responses for a customized experience. Participants hear word audio recordings and select matching images on an iPad screen. It typically takes four minutes and suits ages 3-85, with versions available in English and Spanish. Scoring relies on Item Response Theory, yielding theta scores representing relative ability.

2.2 Oral Reading Recognition Test
Participants are tasked with reading and accurately pronouncing letters and words, with scoring based on correctness. The test employs computer adaptive testing (CAT) and typically takes about three minutes to complete. Scoring relies on Item Response Theory (IRT), producing theta scores representing a participant's overall reading ability, and normative scores are available for the Reading Test.

2.3 Flanker Inhibitory Control and Attention Test
The Flanker task assesses attention and inhibitory control in participants aged 3-85, requiring them to focus on a central stimulus while ignoring surrounding stimuli. Scoring combines accuracy and reaction time using a 2-vector method, resulting in a final score ranging from 0 to 10. If accuracy is 80% or lower, the total score equals the accuracy score; above 80%, it combines accuracy and reaction time scores. For ages 8-85, twenty trials are conducted. The test typically takes about three minutes.

2.4 Dimensional Change Card Sort Test

Dimensional Change Card Sort Test measures cognitive flexibility in participants aged 3-85. It involves matching bivalent test pictures to target pictures, initially based on one dimension (e.g., color) and later switching to the other dimension (e.g., shape). "Switch" trials require participants to change the matching dimension quickly. Scoring combines accuracy and reaction time, using a 2-vector method with scores ranging from 0 to 10. If accuracy is 80% or lower, the total score matches the accuracy score; above 80%, it combines accuracy and reaction time scores. The test takes approximately four minutes to complete and is recommended for ages 3-85.

2.5 Picture Sequence Memory Test

The Picture Sequence Memory Test assesses episodic memory in individuals aged 3-85. Participants recall progressively longer sequences of illustrated objects and activities, with accompanying audio-recorded phrases, displayed on an iPad. Two learning trials involve sequences ranging from 6 to 18 pictures, depending on age. Participants earn points for correctly placing adjacent picture pairs, up to the maximum score, which is one less than the sequence length. The test takes about seven minutes to complete and suits ages 3-85. Scoring involves IRT methodology, converting the number of correctly placed adjacent pairs in each trial to a theta score, offering an estimate of the participant's episodic memory ability. Normative standard scores are provided.

2.6 List Sorting Working Memory Test

This test assesses working memory, requiring immediate recall and sequencing of visually and orally presented stimuli, featuring pictures of foods and animals. Participants are asked to recite the items in size order, first within one dimension (either animals or foods, known as 1-List) and then across two dimensions (foods and animals, referred to as 2-List). The test takes approximately seven minutes and is suitable for ages 7-85, along with normative scores. Scoring involves adding up the total number of correctly recalled and sequenced items on 1-List and 2-List, which can range from 0 to 26. This score is then converted to nationally normed standard scores.

2.7 Pattern Comparison Processing Speed Test

The Speed of Processing test evaluates processing speed by requiring participants to quickly determine whether two pictures displayed side by side are the same or different. Items are presented one pair at a time on an iPad, allowing 85 seconds (excluding any loading time) to respond to as many items as possible, with a maximum of 130 items. The items are intentionally simple to focus on measuring processing speed. The test typically takes around three minutes and is suitable for ages 7-85. Scoring is based on the number of correctly answered items within the 85-second response time, with scores ranging from 0 to 130. These scores are then converted to NIH Toolbox normative standard scores.

Supplemental Results

Amyloid beta and cytokines were assessed at baseline and after exercise regimen.

Table S1. Plasma biomarkers level

(pg/mL)	Pre-Ex	Post-Ex	*p*
Aβ42	107.17 ± 48.86	101.12± 40.45	0.61
Aβ40	340.79 ± 73.91	349.522 ± 66.09	0.57
Aβ (42:40)	0.164± 0.037	0.162 ± 0.034	0.62
Aβ38	695.37 ± 302.79	715.88 ± 305.68	0.26
IFN-γ	8.47 ± 1.14	10.4 ± 3.96	0.63
IL-10	1.92 ± 0.22	1.86 ± 0.23	0.68
IL-2	1.84 ± 0.42	2 ± 0.33	0.5
IL-6	1.55 ± 0.17	1.67 ± 0.2	0.29

Table S1. The plasma biomarker level at baseline (Pre-Ex) and post-exercise (Post-Ex). Aβ: Amyloid beta. IL: Interleukin. IFN-γ: Interferon gamma. Data is Mean ± Standard Error. N=24.

Pearson's correlation coefficient was used to analyze the associations between the change of VO_2max, sleep metrics, BAI, plasma cytokines and cognition functions. All edges between each node were significant related ($p<0.05$). Pearson's *r* were *reported below.*

Table S2A. The associations between the changes of the physiological outcomes

	Features	*Pearson's r*
IL-2 *vs.*	Sleep HR	-0.55
	Rest HR	-0.43
	N3 Delta power	-0.42
	sleep efficiency	0.52
	WASO	-0.54
	Wake	-0.52
	N1	-0.65
	N3	0.47
TNF-α *vs.*	Delta bandpower in N3	0.44
IL-6 *vs.*	sleep efficiency	0.57
	WASO	-0.57
	Wake	-0.57
	N1	-0.57
Aβ38 *vs.*	awakening index	-0.63
	REM	0.55
	N1	-0.49
IFN-γ *vs.*	awakening index	-0.69
	REM	0.66
	N1	-0.54

Table S2B. The associations between the changes of VO_2max and physiological outcomes

	Features	Pearson's *r*	*p* value
**VO_2max** *vs.*	Wake	0.36	0.0688
	REM	0.08	0.6962
	N1	0.004	0.9834
	N2	0.23	0.2506
	N3	0.11	0.591
	NREM	0.35	0.0841
	Cognition Crystallized	0.28	0.1606
	ORR	0.08	0.6829
	PSMT	0.07	0.7335
	PVT	0.35	0.0753
	Delta power	0.16	0.4566

Table S2C. The associations between the changes of BAI and physiological outcomes

	Features	Pearson's *r*	*p* value
**BAI** *vs.*	SHR	0.24	0.2377
	REM	0.06	0.0558
	N1	0.52	0.524
	Cognition Crystallized	0.45	0.4493
	FLD	0.28	0.278
	Cognition Total	0.16	0.1644
	DCCS	0.58	0.5775
	FICA	0.63	0.626
	LSWM	0.38	0.3759
	ORR	0.65	0.6499
	PCPS	0.68	0.6784

Table S2D. The associations between the changes of IL-13 and physiological outcomes

	Features	Pearson's r	*p* value

**IL-13** *vs.*	SHR	-0.04	0.840089
	VO2	-0.04	0.843274
	BAI	-0.60	0.002336
	REM	-0.24	0.260542
	N1	-0.20	0.37175
	N2	0.35	0.101143
	N3	0.55	0.006238
	Cognition Crystallized	-0.29	0.183035
	FLD	0.13	0.546545
	Cognition Total	-0.05	0.814629
	DCCS	0.05	0.803272
	FICA	-0.09	0.687933
	LSWM	0.10	0.662922
	ORR	0.27	0.2129
	PCPS	0.35	0.103005
	PSMT	-0.24	0.274863
	PVT	-0.47	0.024586

Table S2E. The associations between the changes of IL-4 and physiological outcomes

	Features	Pearson's *r*	*p* value
**IL-4** *vs.*	SHR	0.08	0.732565
	VO2	-0.24	0.266667
	BAI	-0.47	0.023961
	Wake	-0.17	0.445639
	REM	-0.35	0.105664
	N1	0.05	0.831171
	N2	0.26	0.230665
	N3	0.22	0.317642
	NREM	0.35	0.106162
	Cognition Crystallized	0.03	0.893263
	FLD	0.23	0.292192
	Cognition Total	0.20	0.363008
	DCCS	-0.08	0.70606
	FICA	0.07	0.75163
	LSWM	0.24	0.275225
	PSMT	-0.34	0.114743
	PVT	-0.25	0.257358

Table S2F. The associations between the changes of IL-8 and physiological outcomes

Features		Pearson's *r*	*p* value
**IL-8** *vs.*	sleep_hr	0.13	0.566883
	VO2	0.02	0.926446
	BAI	0.21	0.340585
	Wake	0.34	0.111813
	REM	-0.20	0.366448
	N1	0.24	0.272439
	N2	-0.17	0.442469
	N3	-0.11	0.606838
	NREM	-0.17	0.425927
	Cognition Crystallized	0.25	0.256303
	FLD	0.08	0.719117
	Cognition Total	0.20	0.359932
	DCCS	0.03	0.907392
	FICA	0.32	0.133865
	LSMT	0.15	0.504691
	ORR	0.13	0.544859
	PCPS	0.14	0.516341
	PSMT	-0.22	0.303554
	PVT	0.21	0.347324

Table S2. The associations between the changes of the physiological outcomes. HR: heart rate. NREM/N: non-rapid eye movement sleep. WASO: wake after sleep onset. TNF-α: tumor necrosis factor alpha. IL: interleukin. Aβ: amyloid beta. IFN-γ: Interferon gamma. Maximal oxygen consumption: VO2max. SHR: sleeping heart rate. FLD: cognition fluid. PSMT: picture sequence memory test. DCCS: dimension change card sort. FICA: flanker Inhibitory control and attention. LSWM: list sort working memory. PCPS: pattern comparison processing speed. ORR: oral reading recognition. PVT: picture vocabulary test. BAI: brain age index. WASO: wake after sleep onset. NREM(N): Non-rapid eye movement. IL-4, 8, and 13: Interleukin-4, 8 and 13. N=24.
